# RAADS-14 Screen: validity of a screening tool for autism spectrum disorder in an adult psychiatric population

**DOI:** 10.1186/2040-2392-4-49

**Published:** 2013-12-09

**Authors:** Jonna M Eriksson, Lisa MJ Andersen, Susanne Bejerot

**Affiliations:** 1 Department of Clinical Neuroscience, Karolinska Institutet, Northern Stockholm Psychiatry, VUB/KogNUS, St. Göran Hospital, SE-112 81, Stockholm, Sweden; 2 Northern Stockholm Psychiatry, VUB/KogNUS, St. Göran Hospital, SE-112 81, Stockholm, Sweden

**Keywords:** Autistic disorder, Asperger syndrome, Adult, Screening, Self-assessment, Rating scale

## Abstract

**Background:**

Autism spectrum disorder (ASD) can be difficult to distinguish from other psychiatric disorders. The clinical assessment of ASD is lengthy, and has to be performed by a specialized clinician. Therefore, a screening instrument to aid in the identification of patients who may have undiagnosed ASD should be useful. The purpose of this study was to develop such a screening instrument.

**Methods:**

Based on the 80 item Ritvo Autism and Asperger Diagnostic Scale-Revised (RAADS-R), we developed a 14 item self-evaluation questionnaire, the RAADS-14 Screen. In total, 135 adults with ASD and 508 psychiatric controls completed the abridged version of the RAADS-R.

**Results:**

The RAADS-14 Screen score was significantly higher in the ASD group than in the control samples, with a median score of 32 for ASD, 15 for attention deficit hyperactivity disorder, and 11 for other psychiatric disorders (*P* < 0.001). A cut-off score of 14 or above reached a sensitivity of 97% and a specificity of 46 to 64%. A factor analysis identified three factors consistent with mentalizing deficits, social anxiety, and sensory reactivity relevant for the diagnosis of ASD. The psychometric properties of RAADS-14 Screen were shown to be satisfactory.

**Conclusions:**

The results of this study indicate that RAADS-14 Screen is a promising measure in screening for ASD in adult psychiatric outpatients.

## Background

Autism spectrum disorder (ASD) is no longer regarded as a rare disorder [1]. Moreover, a high rate of comorbidity with other psychiatric disorders has been observed in adult psychiatric patients with ASD [2,3]. Symptoms in the ASD panorama sometimes overlap with symptoms of mood disorders, anxiety disorders, psychotic disorders, attention deficit hyperactivity disorder (ADHD), or personality disorders, and this may cause diagnostic confusion. Thus, the main diagnostic challenge for psychiatrists today is not to distinguish between individuals with ASD and typically developed individuals, but to identify adult psychiatric patients who may have an undiagnosed ASD for further diagnostic investigation. The clinical procedure for diagnosing ASD typically comprises extensive assessments performed by a team that includes a certified psychologist and a fully trained psychiatrist specialized in diagnosing ASD in adults. This multidisciplinary assessment takes approximately 20 hours to complete, and is based on observations of behaviors, history of childhood symptoms, results from structured and semi-structured interviews with the patient and, when possible, a personal interview with a parent. The patient’s developmental, medical, and psychiatric history is analyzed, and a general physical examination is performed to exclude other possible medical and psychiatric conditions that may contribute to the patient’s current presentation. In adults, self-rating instruments are additionally used. Presumably, the most widely used questionnaires for self-report are the 80-item Ritvo Autism and Asperger Diagnostic Scale-Revised (RAADS-R) [4] and the 50-item Autism Spectrum Quotient (AQ) [5]. The AQ was developed to measure the degree to which adults exhibit cognitive traits typical for autism, whereas the RAADS-R was specifically tailored to assist in the diagnosis of adults within the ASD spectrum by addressing symptoms based on the *Diagnostic and Statistical Manual of Mental Disorders*, Fourth Edition, Text Revision (DSM-IV-TR) for autistic disorder and Asperger’s disorder, and the *International Classification of Diseases*, Tenth Revision (ICD-10) equivalent. Although a self-rating instrument is a cost-effective tool for limiting assessment of individuals with low likelihood for ASD, these two instruments may be considered too lengthy for screening purposes in the clinical setting. Two shorter versions of the AQ were recently launched, and both showed good discriminating properties between ASD and controls in the general population [6,7]. However, user-friendly and psychometrically valid screening instruments for adult ASD tested in psychiatric populations are still lacking. The aim of the present study was to construct such a rating scale, based on the RAADS-R, which would reflect the diagnostic criteria for ASD, and to investigate its properties in a wide range of clinically diagnosed psychiatric outpatients with normal intelligence.

## Method

### Participants

**Figure 1:**
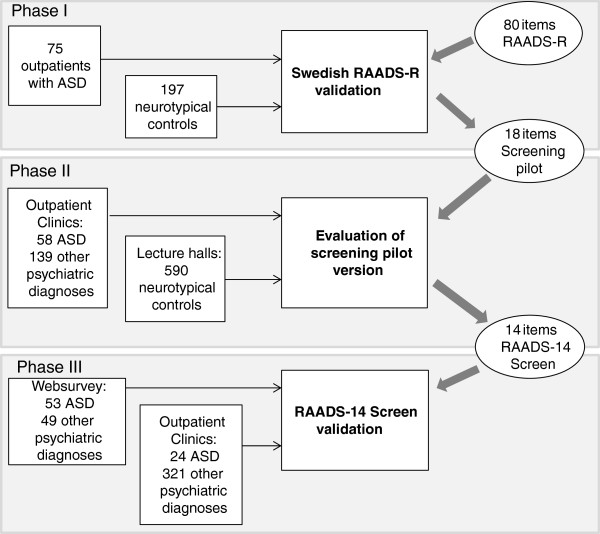
**The three-phase study procedure.** Reduction of items in two phases and a validation of the final RAADS-14 Screen.

A total of 1,233 adults made up of 643 participants with a psychiatric diagnosis and 590 non-psychiatric controls, participated in the study (Table 1). The psychiatric diagnosis participant group included 135 individuals with ASD (that is, autistic disorder, Asperger’s disorder, or pervasive developmental disorder/not otherwise specified, including atypical autism), and 508 participants with ADHD, anxiety disorder, psychotic disorder, borderline personality disorder, or mood disorder. The participants with psychiatric disorders were either psychiatric outpatients (*n* = 541) recruited from 17 Swedish psychiatric clinics, or individuals with a psychiatric diagnosis (*n* = 102) responding via a web-based survey advertised in online communities targeted to people with social anxiety disorder, ADHD, Asperger’s disorder, and mood disorder. The non-psychiatric control group consisted of participants in lectures on mental health, including professionals from education, community, local government, and health sectors (Figure 1). All participants were informed that the study concerned psychiatric diagnostics, and they were asked to fill in a questionnaire anonymously. The patients who completed the form in the psychiatric clinics were all diagnosed by a trained clinician. Swedish psychiatrists adhere to the use of DSM-IV criteria, and follow strict assessment procedures. Before receiving a diagnosis of ASD or ADHD in Sweden, the patient will undergo an extensive assessment process performed by a team of clinicians specialized in neuropsychiatry. The questionnaires were labeled with current diagnoses at completion in the clinic. In the smaller web sample, the participants stated their own diagnosis, and whether they were self-diagnosed or diagnosed at a clinic. Self-diagnosed participants were excluded.

**Table 1:** Sex ratio and age per sample

**Sample**	**n**				**Age, mean ± SD, range**
	**Male**	**Female**	**Unknown**	**Total**	
Phase II					
ASD^a,b^	31	22	5	58	33.7 ± 11.5, 16 to 70
ADHD	16	23	4	43	37.0 ± 12.2, 19 to 64
Other psychiatric disorders^c^	41	50	4	95	35.6 ± 12.4, 18 to 77
Mood disorders^d^	17	13	1	31	
Anxiety disorders^e^	11	22	1	34	
Psychotic disorders^f^	11	7	2	20	
Borderline personality disorder	8	19	1	28	
Non-psychiatric	105	400	85	590	45.0 ± 10.9, 19 to 66
Phase III					
ASD^g^	32	43	2	77	35.2 ± 10.9, 16 to 58
ADHD	11	162	28	301	32.6 ± 12.0, 17 to 68
Other psychiatric disorders	23	45	1	69	32.8 ± 9.8, 18 to 57
Mood disorders	9	19		28	
Anxiety disorders	7	26		33	
Psychotic disorders	5	5		10	
Borderline personality disorder	3	7		10	

^a^Coexisting psychiatric disorder in *n* = 23 (40%).

^b^ASD includes autistic disorder, Asperger’s disorder and atypical autism.

^c^In the ‘other psychiatric disorders group’, specific diagnoses are stated; however, the sums of reported diagnoses do not add up to the number of patients in the group because these patients may have co-occurring diagnoses.

^d^Mood disorders include major depression and bipolar I and II.

^e^Anxiety disorders include obsessive-compulsive disorder, generalized anxiety disorder, social anxiety disorder and post-traumatic stress disorder.

^f^Psychotic disorders include brief psychosis, schizoaffective disorder and schizophrenia.

^g^Coexisting psychiatric disorder in *n* = 45 (58%).

### Materials

The questionnaire included items derived from the RAADS-R and additional questions about age, sex, and psychiatric diagnoses.

### Ritvo Autism and Asperger Diagnostic Scale-Revised

The RAADS-R comprises 80 statements assessing autistic traits closely matching the diagnostic criteria in the DSM-IV-TR, with the addition of sensory motor symptoms. The response alternatives to each statement are given on a four-point Likert scale (ranging from 0 to 3) indicating duration of each symptom (3 = ‘true now and when I was young’, 2 = ‘true only now’, 1 = ‘true only when I was younger than 16’ and 0 = ‘never true’). Seventeen statements are reversely formulated in order to limit effects of response bias [8]. The items correspond to the four domains of social relatedness, circumscribed interests, language, and sensory motor symptoms. Item scores are summed to a total score, which has shown good discriminative power for ASD [4]. The RAADS-R was translated into Swedish, and validated in a Swedish population consisting of 75 participants with ASD and 197 non-ASD controls [9].

### Procedure

The objective was to minimize the number of items required from the RAADS-R, while preserving as much as possible of its discriminative power and broad symptomatology assessment, in order to obtain a short questionnaire for ASD screening in the psychiatric population. Starting with the 80 items in the RAADS-R, 14 items were selected and tested for good psychometric properties in a three-phase process (Figure 1).

#### Phase I

Based on the results from the Swedish validation of RAADS-R [9], 18 items that differentiated ASD participants from non-psychiatric controls were selected. In order to maintain content validity [10], items were selected in proportions corresponding to the original RAADS-R domains, in a number that would fit into a one-page questionnaire. Within each of the four domains in the RAADS-R, the items that best differentiated between the ASD group and the control group, and at the same time were good representatives of the domain, were determined by calculation of a discrimination index for each item. This was defined as the product of 1) the Pearson effect sizes of the difference in means between the ASD group and the control group for each item, and 2) a corrected item-total correlation; that is, the strength of the correlation of each item with all the other items in that item’s domain.

#### Phase II

The 18 item pilot version was tested on 196 psychiatric outpatients, with (*n* = 58) and without (*n* = 138) ASD, and 590 non-psychiatric participants. Four items that failed to identify ASD in the psychiatric population were removed. We reordered the items, putting the five items with the best discriminating properties at the beginning of the questionnaire to allow for the possibility of a short form screener. The remaining items were mixed with respect to domain. Item numbers are given according to the final order (Table 2). Two senior psychiatrists and a senior psychologist independently investigated the 14 items of the final RAADS-14 Screen for congruency with the ASD items in the proposed fifth edition of the DSM (DSM-5). If the clinicians’ categorization on any item differed, they reached a consensus after discussion.

**Table 2:** The RAADS-14 Screen

Please choose one of the following alternatives:					
This is true or describes me *now and when I was young*.					
This was true or describes me *only now* (refers to skills acquired).					
This was true *only when I was young* (16 years or younger).					
This was *never true and never described me*.					
Please answer the questions according to what is true for you. Check only one column per statement!					
	Some life experiences and personality characteristics that may apply to you	True now and when I was young	True only now	True only when I was younger than 16	Never true
1	It is difficult for me to understand how other people are feeling when we are talking				
2	Some ordinary textures that do not bother others feel very offensive when they touch my skin				
3	It is very difficult for me to work and function in groups				
4	It is difficult to figure out what other people expect of me				
5	I often don’t know how to act in social situations				
6*	I can chat and make small talk with people				
7	When I feel overwhelmed by my senses, I have to isolate myself to shut them down				
8	How to make friends and socialize is a mystery to me				
9	When talking to someone, I have a hard time telling when it is my turn to talk or to listen				
10	Sometimes I have to cover my ears to block out painful noises (like vacuum cleaners or people talking too much or too loudly)				
11	It can be very hard to read someone’s face, hand, and body movements when we are talking				
12	I focus on details rather than the overall idea				
13	I take things too literally, so I often miss what people are trying to say				
14	I get extremely upset when the way I like to do things is suddenly changed				

*reversely scored item.

#### Phase III

The discriminatory properties of the RAADS-14 Screen were tested in 447 participants reporting ASD, ADHD, or another other psychiatric disorder.

A total of 89 individuals from phase II and III were excluded for: 1) failure to report a confirmed psychiatric diagnosis (*n* = 40); 2) report of a psychiatric diagnosis if the person had been recruited as a non-psychiatric control (*n* = 11); 3) indication of not having read the statements properly (that is, all items checked identically without notice of a reversed item) (*n* = 35); 4) more than four missing items (*n* = 2); or 5) a diagnosis of mental retardation (*n* = 1). Included participants were categorized into six diagnostic groups and one group of non-psychiatric controls, as shown in Table 1.

### Statistics

All analyses were computed using SPSS 21.0.0.0. Similarities between distributions were tested with the independent samples Kruskal-Wallis test. Owing to the non-normal nature of the data, all differences between items and scores according to group and gender were tested with the Mann–Whitney *U*-test, with effect size calculated as $r=Z/\sqrt{N}$[11] (the effect is considered large for *r* ≥ 0.5, medium for 0.5 > *r* ≥ 0.3, and small for 0.3 > *r* ≥ 0.1).

Discriminatory power was assessed using a receiver operating characteristic (ROC) curve. For each score, the rate of true positives (sensitivity) is plotted against the rate of false positives (1 minus specificity). The area under the curve (AUC) is a measure of the discriminatory power. An AUC greater than 0.7 is considered acceptable. Aiming to achieve good screening properties with a high sensitivity, the cut-off score was selected by choosing the lowest score corresponding to a true positive rate of 93% or greater in the combined phase II and phase III ASD samples.

### Ethics approval

All participants provided their informed consent before completing the questionnaire. The questionnaires were regarded as research material and were responded to anonymously. The responses were coded in accordance with an established procedure. The study was approved by the Ethics Committee of Karolinska Hospital (Dnr 2010/1343-31/3).

## Results

### Phase I: construction of a pilot version

The RAADS-R items with the highest discriminating indexes from each of the four domains were: circumscribed interests (items 9, 24, 30, 32), language (items 27, 58), social relatedness (items 5, 17, 25, 45, 55, 60, 64, 76) and sensory motor (items 29, 42, 57, 73).

### Phase II: evaluation of an 18 item pilot version

#### Construct validity and reliability

An exploratory factor analysis was conducted to assess the construct validity. Although the original scale has a four-factor structure, the selection of the most discriminatory items from each factor made patients with ASD endorse almost all items, resulting in a one-factor solution. However, if the ASD group was excluded, a three-factor solution emerged, with a meaningful interpretation. The three eigenvalues, selected from the scree plot of the analysis of the collapsed psychiatric and non-psychiatric control groups, yielded three principal factors, respectively explaining 32.5%, 8.5%, and 6.4% of the total variance. Factor 1 was interpreted as a domain regarding ‘mentalizing deficits’ and factor 2 was interpreted as ‘sensory reactivity’, while factor 3 was named ‘social anxiety’ (Table 3). The reliability was excellent (*α* ≥ 0.92) for the full 18 item scale and satisfactory (*α* > 0.7) for all three principal factors in the full sample. The original four-factor structure of RAADS-R was also tested with Cronbach’s alpha and showed good internal consistencies (*α* > 0.8) for three domains, and poor consistency (*α =* 0.42) for the language domain.

**Table 3:** **The RAADS-14 Screen domains related to the Ritvo Autism and Asperger Diagnostic Scale-Revised (RAADS-R) domains and the *Diagnostic and Statistical Manual of Mental Disorders*, Fifth Edition (DSM-5) criteria for autism spectrum disorder (ASD)**

**RAADS-14 screen domain**	**Items**	**DSM-5 criteria**	**Original RAADS-R domain**
Mentalizing deficits	13. I take things too literally, so I often miss what people are trying to say	A1	Language
	1. It is difficult for me to understand how other people are feeling when we are talking	A2	Social relatedness
	9. When talking to someone, I have a hard time telling when it is my turn to talk or to listen	A1	Social relatedness
	4. It is difficult to figure out what other people expect of me	A2	Social relatedness
	11. It can be very hard to read someone’s face, hand, and body movements when we are talking	A2	Social relatedness
	12. I focus on details rather than the overall idea	B3	Circumscribed interests
	14. I get extremely upset when the way I like to do things is suddenly changed	B2	Circumscribed interests
Social anxiety	3. It is very difficult for me to work and function in groups	A1 (A3)	Social relatedness
	5. I often don’t know how to act in social situations	A1	Social relatedness
	6. ^a^I can chat and make small talk with people	A1	Language
	8. How to make friends and socialize is a mystery to me	A3 (A1)	Social relatedness
Sensory reactivity	2. Some ordinary textures that do not bother others feel very offensive when they touch my skin	B4	Sensory motor
	7. When I feel overwhelmed by my senses, I have to isolate myself to shut them down	B4	Sensory motor
	10. Sometimes I have to cover my ears to block out painful noises (like vacuum cleaners or people talking too much or too loudly)	B4	Sensory motor

^a^Reversed statement.

#### Distribution of scores

**Figure 2:**
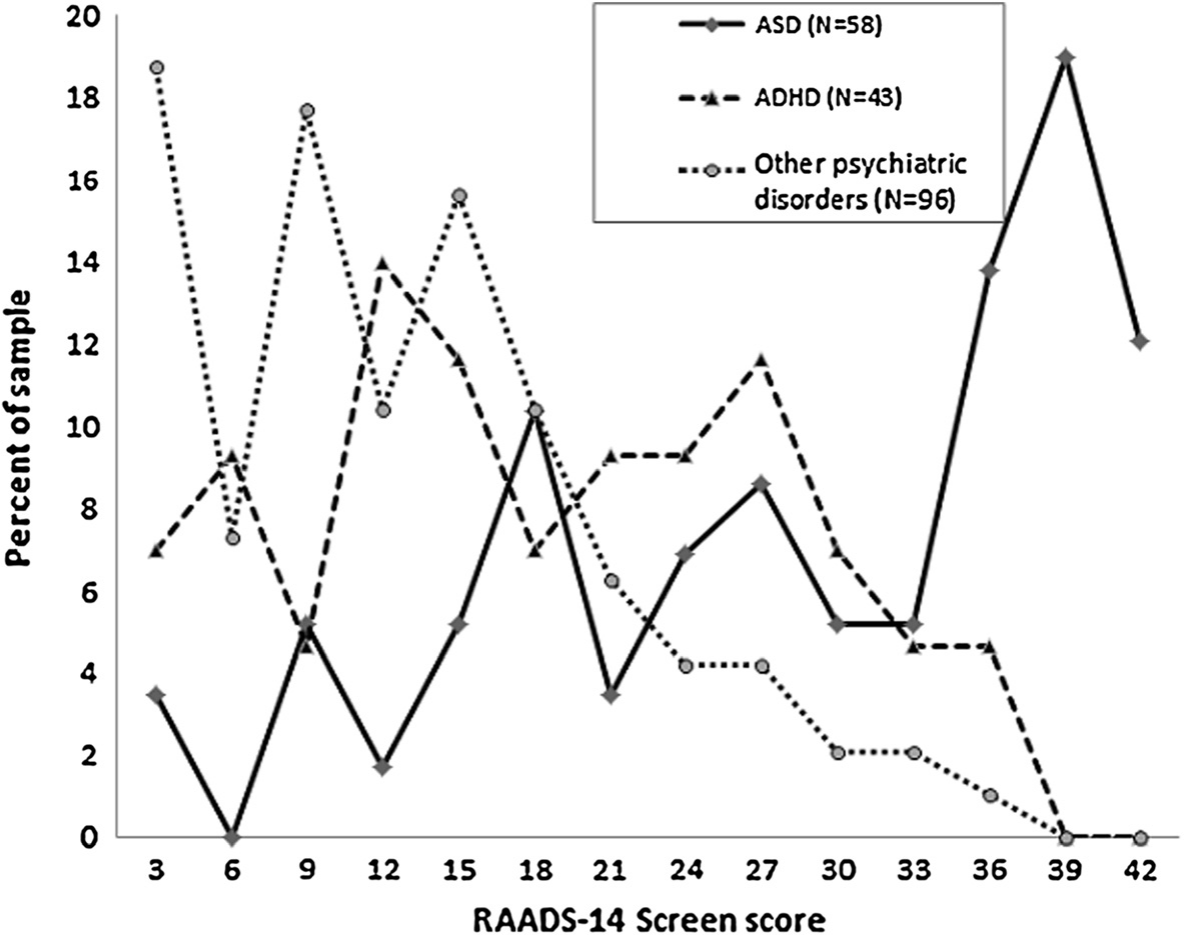
**Distributions of RAADS-14 Screen score in the phase II psychiatric samples.** ASD consists of autistic disorder, Asperger’s disorder and atypical autism. ADHD consists of participants with ADHD but no ASD diagnosis. ‘Other psychiatric disorders’ consists of participants with a psychiatric diagnosis not including ASD or ADHD.

To study the distributions of the 18 item questionnaire scores in different groups of psychiatric diagnoses, only individuals who had reported one single diagnosis were included. Distributions of scores for the six psychiatric groups (ASD, ADHD, anxiety disorders, mood disorders, psychotic disorders, and borderline personality disorder) were compared and showed three different distribution patterns, being left-skewed in the ASD group and right-skewed in the non-psychiatric group, and all psychiatric subgroups except the ADHD group (Figure 2). Thus, the results of the participants with a psychiatric diagnosis are henceforth presented for the three samples: ASD, ADHD without ASD, and other psychiatric disorders (OPD) without ASD or ADHD.

### Item selection for RAADS-14 Screen

A ROC analysis of each item tested the power of ASD discrimination in the psychiatric group. The four items with the lowest discriminatory power were removed from the questionnaire (that is: ‘I can’t tolerate things I dislike (like smells, textures, sounds, or colors)’; ‘It is difficult for me to start and stop a conversation. I need to keep going until I am finished’; ‘I get highly confused when someone interrupts me when I am talking about something I am very interested in’; and ‘Others consider me odd or different’).

### Phase III: validation of psychometric properties of RAADS-14 Screen

#### Reliability and discriminatory powers of RAADS-14 Screen

The reliabilities of the RAADS-14 Screen and the three domains obtained in phase II were calculated for each group (Table 4). In the combined phase II and phase III sample, the items in the RAADS-14 Screen showed excellent internal consistency (*n* = 1,233, *α* = 0.9) for the full scale.

**Table 4:** Scores on the RAADS-14 Screen, differences from the autism spectrum disorders (ASD) sample, and internal consistency

**Phase**	**n**	**Mean ± SD**	**RAADS-14 Screen score**			**Internal consistency, *α***			
			**Median (range)**	**M-W *U (z)***^**a**^	***r***^**b**^	**F1**^**c**^	**F2**^**d**^	**F3**^**e**^	**RAADS-14 Screen**
Phase II									
ASD	58	27.9 ± 11.5	30 (0–42)	–	–	0.84	0.67	0.63	0.88
ADHD	43	17.5 ± 9.5	18 (1–36)^f^	596.0 (-4.5)	0.68	0.80	0.42	0.51	0.77
Other psychiatric disorders	95	11.9 ± 8.3	12 (0–39)^f^	772.5 (-7.5)	0.57	0.75	0.54	0.57	0.78
Non-psychiatric controls	590	3.9 ± 4.6	3 (0–29)^f^	1528 (-11.6)	0.48	0.65	0.32	0.53	0.70
Total sample	792	7.4 ± 9.3	4 (0 – 42)	–	–	0.86	0.64	0.76	0.90
Phase III									
ASD	77	30.8 ± 8.6	32 (8–42)			0.75	0.62	0.62	0.80
ADHD	301	15.4 ± 9.3	15 (0–42)^f^	2824 (-10.3)	0.59	0.73	0.49	0.62	0.79
Other psychiatric disorders	69	12.6 ± 9.3	11 (0–39)^f^	467.5 (-8.6)	0.71	0.75	0.40	0.76	0.84
Total sample	457	17.4 ± 11.0	16 (0 – 42)	–		0.80	0.58	0.73	0.86

^a^Mann–Whitney *U*-test.

^b^Effect size.

^c^Mentalizing Deficits items: 1, 4, 9, 11, 12, 13, 14.

^d^Sensory Reactivity items: 2, 7, 10.

^e^Social Anxiety items: 3, 5, 6, 8.

^f^*P* < 0.001.

**Figure 3:**
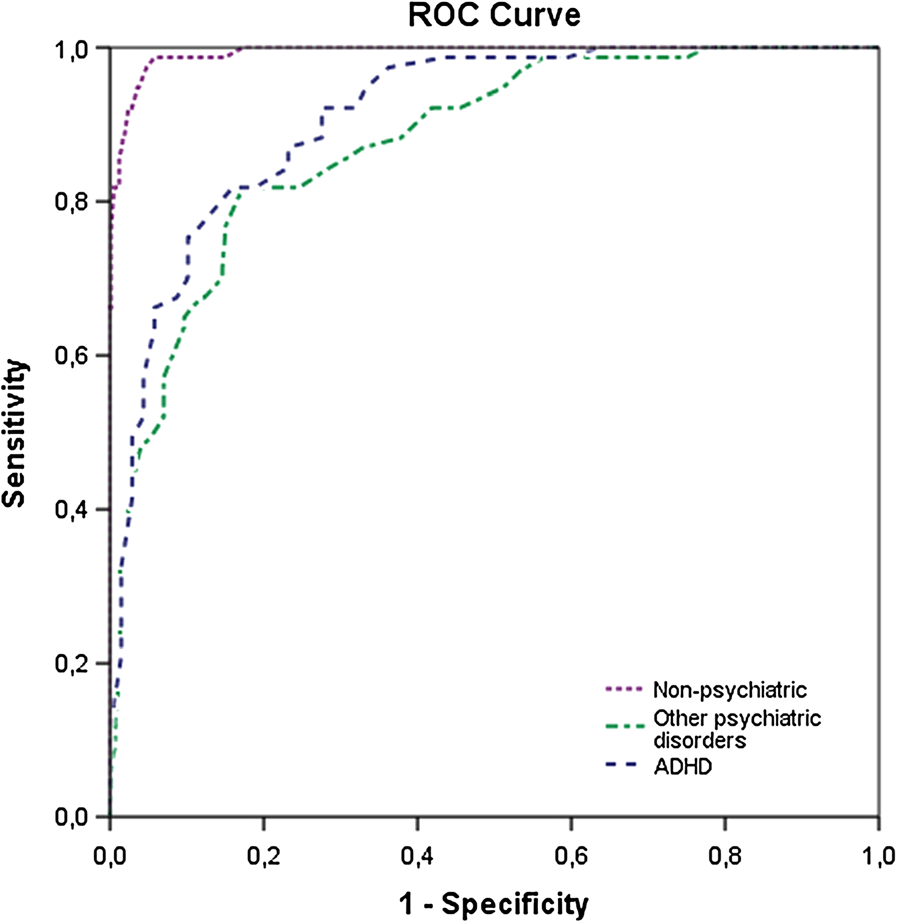
**Discriminating power of the RAADS-14 Screen.** Receiver operating characteristic (ROC) curves for phase III samples of the autism spectrum disorder (ASD) group (*n* = 77) together with: 1) the Other psychiatric disorders group (*n* = 69, dashed line), area under the curve (AUC) = 0.91; 2) the attention deficit hyperactivity disorder (ADHD) group (*n* = 301, dashed-dotted line), AUC = 0.88; and 3) the non-psychiatric group (n = 590, dotted line), AUC = 0.99.

**Figure 4:**
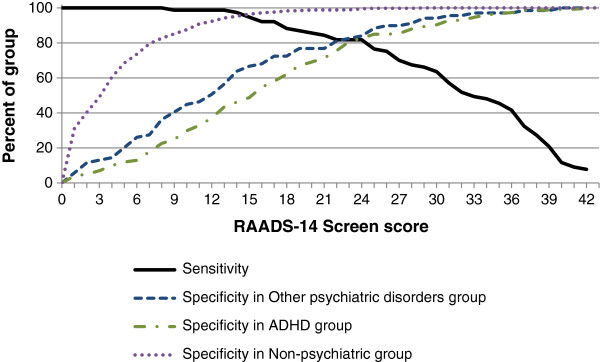
**Operating characteristics of the RAADS-14 Screen.** Sensitivity and specificity for various threshold scores for 77 individuals with autism spectrum disorder (ASD) compared with 301 individuals with attention deficit hyperactivity disorder (ADHD) but no ASD (dashed-dotted line), 69 individuals with Other psychiatric disorder, not including ASD or ADHD (dashed line), and 590 non-psychiatric individuals (dotted line).

The discriminatory power was studied in the phase III samples. A ROC curve comparing the results of all participants with ASD (*n* = 77) and non-ADHD psychiatric controls (*n* = 69) yielded an AUC of 0.91, while the ASD and ADHD (*n* = 301) comparison yielded an AUC of 0.88 (Figure 3). To evaluate the discriminatory power in the non-psychiatric population, the ASD group from phase III was also tested against the non-psychiatric group (*n* = 590) from phase II, yielding an AUC of 0.99. A cut-off point of 14 or greater out of a maximum of 42 points yielded a 0.97 sensitivity and a specificity of 0.46 in the ADHD sample, 0.64 in the OPD sample, and 0.95 in the non-psychiatric control group (Figure 4). Of the 135 individuals diagnosed with ASD in phase II and III, 9 scored below the cut-off point on the RAADS-14 Screen, including 2 who scored zero.

The discriminatory power of the first five items was moderately good for both the ADHD sample (AUC = 0.86) and the OPD sample (AUC = 0.88), and excellent for the non-psychiatric sample (AUC = 0.98). A cut-off point of 4 or greater from a maximum of 15 points yielded a sensitivity of 0.93 and a specificity of 0.45 in the ADHD sample, 0.49 in the OPD sample, and 0.90 in the non-psychiatric sample.

#### Comparison of RAADS-14 Screen scores between diagnostic groups

**Figure 5:**
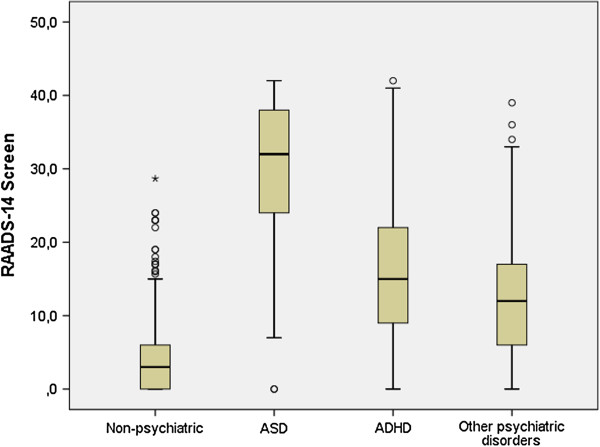
**Box plot of the RAADS-14 Screen score in the samples of phase III.** The bottom and top of the box indicate the 25th and 75th percentiles, respectively. The whiskers extend to 1.5 times the height of the box (or to the minimum or maximum values). Circles denote values outside this range. Autism spectrum disorder (ASD) consists of autistic disorder, Asperger’s disorder, and atypical autism. Attention deficit hyperactivity disorder (ADHD) consists of participants with ADHD but no ASD diagnosis. ’Other psychiatric disorders’ consists of participants with a psychiatric diagnosis not including ASD or ADHD.

The total RAADS-14 Screen scores were similarly distributed between the phase II and phase III samples for the ASD group and the ADHD and OPD control groups. Furthermore, no statistically significant difference was found in the comparison between the clinical and web samples scores and distributions for the three psychiatric groups. The comparison of scores showed that the RAADS-14 Screen total score was significantly higher (*P* < 0.001) in the ASD group than in the two control groups (median: ASD, 32; ADHD, 15; OPD, 11) (Figure 5), and the effect sizes indicated a large effect. Similarly, the effect sizes were large in all four subgroups within the OPD sample, that is, anxiety disorders, mood disorders, borderline personality disorder, and psychotic disorders. Moreover, the ASD sample scores also differed from the groups of patients with psychiatric disorders when analyzes against the subgroups of patients with various psychiatric disorders (obsessive-compulsive disorder, generalized anxiety disorder, social anxiety disorder, post-traumatic stress disorder, major depression, bipolar I, bipolar II, brief psychosis, schizoaffective disorder, and schizophrenia) in the combined phase II and phase III samples. Furthermore, at item level, all 14 scores were lower (*P* < 0.002) in both the ADHD and OPD samples. Only in patients with social anxiety disorder were there two items that did not differ from ASD (items 5 and 6).

#### Gender differences

In the ASD, ADHD, and non-psychiatric samples, females obtained significantly higher scores than males in the sensory reactivity domain, suggesting higher sensory sensitivity in females, (Table 5). Conversely, non-psychiatric males scored higher than females in the mentalizing deficits and social anxiety domains.

**Table 5:** Scores of the RAADS-14 Screen and subscales for males and females

**Sample**^**a**^	**Sex**	**n**	**RAADS-14-Screen**	**Mentalizing deficits**	**Social anxiety**	**Sensory reactivity**
			**Median (range)**			
Non-psychiatric	M	105	3 (0 to 19)	1 (0 to 13)^b^	1 (0 to 8)^c^	0 (0 to 6)
	F	400	2.5 (0 to 29)	0 (0 to 19)	0 (0 to 10)	0 (0 to 9)^b^
ASD	M	64	30 (0 to 42)	15 (0 to 21)	9.7 (0 to 12)	6 (0 to 9)
	F	66	34 (9 to 42)	18 (3 to 21)	9 (0 to 12)	8 (3–9)^d^
ADHD	M	127	15 (0 to 36)	7 (0 to 21)	3 (0 to 12)	3 (0 to 9)
	F	185	15 (0 to 42)	8 (0 to 21)	3 (0 to 12)	3 (0 to 9)^d^
Other psychiatric disorders	M	64	11.5 (0 to 33)	4 (0 to 21)	4 (0 to 11)	2 (0 to 9)
	F	95	12 (0 to 39)	5 (0 to 21)	2 (0 to 12)	3 (0 to 9)

ADHD, attention deficit hyperactivity disorder; ASD, autism spectrum disorder.

^a^Samples selected from phases II and III combined.

^b^*P* < 0.05, ^c^*P* < 0.01, ^c^*P* < 0.001, differences are indicated in the sex with the higher score.

## Discussion

This study set out to develop and test a short self-evaluation questionnaire for assessing ASD in adults without intellectual disability. The RAADS-14 Screen, derived from the RAADS-R, showed good psychometric properties, and takes only a few minutes to complete, compared with up to 1 hour for the original RAADS-R. Notably, individuals with ASD scored high in all three domains comprising the RAADS-14 Screen, showing mentalizing deficits, increased social anxiety, and sensory oversensitivity, thus capturing characteristics typical of ASD. Thus we suggest the RAADS-14 Screen is a useful tool for screening adult psychiatric outpatients for an unrecognized ASD, which often exists comorbidly with other disorders such as major depression, obsessive-compulsive disorder, ADHD, and various anxiety disorders [2,3].

The RAADS-14 Screen shows excellent discrimination abilities in the non-psychiatric population. Furthermore, because of its established capability to distinguish ASD from OPD, which may obscure the clinical picture, the RAADS-14 Screen also has extended screening utility. In the final sample, a cut-off score of 14 or above in the RAADS-14 Screen correctly identified 97% of the participants with ASD and excluded an incorrect ASD diagnosis in 46 to 64% of patients with ADHD or OPD. However, as described in the Introduction, some patients with OPD may also have undiagnosed ASD; it is therefore likely that some participants in the psychiatric control groups should have been diagnosed with ASD. If this is the case, the aforementioned specificity of 46 to 64% may be an underestimate.

Although the short versions of AQ are equally good at discrimination in the non-psychiatric population, they have not been tested as to whether they are useful for distinguishing ASD from overlapping psychiatric disorders. Moreover, the different versions of AQ aim to assess autistic traits in the general population, whereas the RAADS-14 Screen aims to spot ASD. Evaluations of the abridged versions of AQ have resulted in mean scores reaching around 50% of the total AQ score in a normal population sample for the 28 item version, and 28% in the 10 item version [6,7], whereas healthy controls only reach a mean score of 9% of the total RAADS-14 Screen score. In addition, sensory reactivity, included in the diagnostic criteria for DSM-5, is not included in the AQ 28 item version, and comprises only one item in the AQ-10 version, compared with three items in the RAADS-14 Screen.

The first five items in the RAADS-14 Screen could serve as initial rapid screen. A cut-off score of 4 or above out of a possible maximum score of 15 correctly diagnosed 93% of the patients with ASD, while 45 to 49% of the psychiatric controls were (presumably) correctly excluded as having ASD. These first items may provide sufficient information when time is very limited, or when the patient is reluctant or unable to respond to many questions.

Out of 135 patients with ASD in phases II and III, 9 scored below the cut-off for ASD, including 2 individuals that scored zero points. As it seems highly unlikely that a person with ASD would not have any of the core symptoms mentioned in the questionnaire, other reasons such as lack of insight, misinterpretation, or unwillingness to endorse any of the statements are more likely. This highlights the benefits of having a clinician present during the completion of the questionnaire, both for explaining the statements to the patient if needed, and for assessing the congruity between the observed symptoms and the responses in the questionnaire.

### Endorsement patterns in the different diagnostic groups

For validity, based on the magnitude, pattern, and significance of difference of the items between the ASD group and the clinical control groups, the RAADS-14 Screen has good validity for criteria and convergence. With regard to the ADHD group, the differences were smaller, although still clinically meaningful. This lower difference could be an indicator of some items being discriminators for early-onset neurodevelopmental disorders in general, rather than ASD specifically. It could also be due to the fact that many individuals with ADHD have co-existing autistic traits [12,13].

The items in the social anxiety domain overlapped with symptoms suggestive of social anxiety disorder. The responses to two of these items (I often don’t know how to act in social situations’ (item 5) and ‘I can chat and make small talk with people’ (item 6)) were similar between the patients with social anxiety disorder and the ASD group. People with ASD exhibit deficits in social skills, and often experience peer rejection, which may induce social anxiety. In addition, a large difference in the total RAADS-14 Screen scores meant that the ASD and social anxiety disorder groups were clearly distinguishable. Compared with patients with other anxiety disorders, the ASD group scored higher on all the social anxiety items, indicating to the importance of taking all domains into consideration when using the RAADS-14 Screen.

### Factor structure

Based on Cronbach’s alpha, the internal consistency of the factors in the identified three-factor structure exceeded 0.7 for all clinical groups, the recommended minimum level for group comparisons in clinical studies [14]. These factors were characterized as mentalizing deficits, sensory reactivity, and social anxiety. In the DSM-5 for ASD [15], the A criteria ‘Persistent deficits in social communication and social relatedness across contexts’ include descriptions that are congruent with eight items in the mentalizing deficits and social anxiety domains on the RAADS-14 Screen. The B criteria ‘Restricted, repetitive patterns of behavior, interests, or activities’ correspond with a total of five items in the mentalizing deficits and the sensory reactivity domains. Stereotypies is the only DSM-5 item that is not covered by the RAADS-14 Screen; however, self-reported stereotypies may not be a valid option for estimating their presence, because individuals are often unaware of having them. Together, these results suggest that the RAADS-14 Screen will be useful when diagnosing people according to the DSM-5 criteria for ASD (Table 3).

### Construct validity

The mentalizing deficit domain of the RAADS-14 Screen includes all the items from the original RAADS-R circumscribed interests’ domain, half of the items from the social relatedness domain, and one language item. The items from the circumscribed interests RAADS-R domain were all related to an ability to change from an internal to an external focus, which fits well into a domain of mentalizing deficits. In addition, four of the social relatedness items, relating to understanding social codes and reading body language, and one language item assessing the mentalizing skill of making a distinction between the literal and intended meanings of sentences, fit nicely into the new mentalizing deficit domain. Another four social relatedness items and one language item describe feelings of social awkwardness and inabilities, thus they formed the second domain, social anxiety. Finally, the sensory motor domain of the RAADS-R was not compromised by the new factor structure, but none of the pure motor function items in the RAADS-R was sufficiently discriminative of ASD to be included in the RAADS-14 Screen. Although poor gross motor skills are common in ASD, it is by no means specific to any psychiatric disorder, and children with emotional, behavioral, and pervasive developmental disorders often exhibit gross motor problems [16].

### Gender difference

In the non-psychiatric disorders group, females had fewer mentalizing deficits and social anxiety than males. Notably, a reverse trend was found in the ASD group, with females scoring higher than males in the mentalizing deficits domain. Both findings are consistent with the Swedish RAADS-R validation study [9]. The former findings support the extreme male brain theory for autism, that, in general, males have more cognitive autistic traits than females [17]. The reason for poorer mentalizing skills in females with ASD compared with males is intriguing. Possibly, females with ASD have greater insight into their behaviors, and thus endorse autistic symptoms more readily than the males. The scores of the psychiatric controls in the mentalizing deficits and social anxiety domains were independent of gender. However, females scored higher than males in the sensory reactivity domain, across all groups, supporting the gender differences in sensitivity to noise and touch that have been reported in earlier studies [18,19].

Some methodological limitations should be noted. Selection bias is always a crucial issue in clinical research. In order to include a representative population of patients, a desirable design for a validity study would be to collect data from all psychiatric patients visiting a clinic in a certain time period; however, this design does not protect against selection bias. For prospective research studies, informed written consent from the patient is a prerequisite, and this may result in attrition due to a reluctance to participate [20]. Further selection bias could be related to the variability in frequency of consultations; some patients rarely visit the clinic even if they are severely ill, whereas others are frequent visitors. In the present study, selection bias is difficult to estimate because the collection of patient data was performed by a number of clinicians across the country, and through web pages directed towards people with psychiatric diagnoses. However, the option to respond via a web survey may enable responses from patients who, for different reasons (such as poor executive skills), rarely visit the clinic, thus possibly improving the representativeness of our sample. The inclusion of a number of highly specialized psychiatric clinics was also chosen to broaden the spectrum of patients compared with a general psychiatric clinic. Another limitation is the lack of confirmatory assessments to confirm or exclude diagnoses, but Swedish clinical practice is to use reliable diagnostic instruments. The truthfulness of the web respondents is supported by the fact that their scores were not different from those of the clinical samples. The 16 suspected outliers in the non-psychiatric group indicate that some of these subjects actually have an undiagnosed psychiatric disorder. Although a weakness of the study, it makes it plausible that the specificity in this group is even better. Although the ASD sample was not matched for gender, age, or intelligence in the comparison samples, the age distributions of all three psychiatric samples were roughly the same. Moreover, 10% of the participants did not state their gender; this may have had an influence on the gender difference results. Finally, as the DSM-5 was introduced after the completion of the current study, the RAADS-14 Screen was inevitably validated in psychiatric patients diagnosed according to the DSM-IV-TR. However, all items included in the RAADS-14 Screen are applicable for the DSM-5 ASD criteria, suggesting a utility for the RAADS-14 Screen beyond DSM-IV. The RAADS-14 Screen print-out version with instructions to the clinician is available as an Additional file 1.

## Conclusions

The current study supported the construct validity, convergent validity, and internal consistency reliability in the three-factor structure of the RAADS-14 Screen. This abridged version of the RAADS-R is a promising measure for screening for ASD in psychiatric outpatients, in whom comorbidities may confuse the diagnostic process. The higher the RAADS-14 Screen score, the more reason to perform a full evaluation. This self-rating questionnaire takes only a few minutes to complete, identifies most patients with ASD while excluding approximately 50% of non-ASD patients, using the suggested cut-off point of 14. A rapid clinician review of the questionnaire together with the patient may clarify ambiguities, and further improve the efficacy of the screening.

## Abbreviations

ADHD: Attention deficit/hyperactivity disorder; ASD: Autism spectrum disorder; AQ: Autism spectrum quotient; AUC: Area under the curve; RAADS-R: Ritvo autism and asperger diagnostic scale-revised; ROC: Receiver operating characteristics.

## Competing interests

The authors declare that they have no competing interests.

## Authors’ contributions

LA participated in project design, and performed the phase I statistical analysis and selection of items. JE participated in project design, performed phase II and III statistical analysis, and wrote the manuscript. SB formulated the study aims, coordinated the collection of data, participated in project design, and helped draft the manuscript. All authors read and approved the final manuscript.

## Supplementary Material

Additional file 1 RAADS-14 Screen. Click here for file
