# Supplementary material for: RAADS-14 Screen: validity of a screening tool for autism spectrum disorder in an adult psychiatric population
Source: Mol Autism. 2013 Dec 9;4:49. doi: 10.1186/2040-2392-4-49 (PMC3907126; doi:10.1186/2040-2392-4-49)
Supplement: Additional file 1 — RAADS-14 Screen. [file 2040-2392-4-49-S1.pdf]

RAADS-14 Screen

Name:	Patient ID:
Date:	Clinician:

Please choose one of the following alternatives:
This is true or describes me now and when I was young.
This was true or describes me only now (refers to skills acquired).
This was true only when I was young (16 years or younger).
This was never true and never described me.

Please answer the questions according to what is true for *you*. Check only one column per statement!

	Some life experiences and personality characteristics that may apply to you	True now and when I was young	True only now	True only when I was younger than 16	Never true
1.	It is difficult for me to understand how other people are feeling when we are talking.	☐	☐	☐	☐
2.	Some ordinary textures that do not bother others feel very offensive when they touch my skin.	☐	☐	☐	☐
3.	It is very difficult for me to work and function in groups.	☐	☐	☐	☐
4.	It is difficult to figure out what other people expect of me.	☐	☐	☐	☐
5.	I often don’t know how to act in social situations.	☐	☐	☐	☐
6.*	I can chat and make small talk with people.	☐	☐	☐	☐
7.	When I feel overwhelmed by my senses, I have to isolate myself to shut them down.	☐	☐	☐	☐
8.	How to make friends and socialize is a mystery to me.	☐	☐	☐	☐
9.	When talking to someone, I have a hard time telling when it is my turn to talk or to listen.	☐	☐	☐	☐
10.	Sometimes I have to cover my ears to block out painful noises (like vacuum cleaners or people talking too much or too loudly).	☐	☐	☐	☐
11.	It can be very hard to read someone’s face, hand, and body movements when we are talking.	☐	☐	☐	☐
12.	I focus on details rather than the overall idea.	☐	☐	☐	☐
13.	I take things too literally, so I often miss what people are trying to say.	☐	☐	☐	☐
14.	I get extremely upset when the way I like to do things is suddenly changed	☐	☐	☐	☐

RAADS-14-Screen is an abridged version of Ritvo Autism and Asperger Diagnostic Scale-Revised (RAADS-R). Cite: Eriksson JM, Andersen MJ, Bejerot S. RAADS-14 Screen: validity of a screening tool for Autism Spectrum Disorder in an adult psychiatric population. *Molecular Autism* 2013; 4:49.

RAADS-14 Screen

Scoring:

- All items (except item 6 which is reversed and therefore marked with *) range from 3 to 0.
- The score range from a minimum of 0 to a maximum of 42.

RAADS-14 Screen contains three subdomains:

- Mentalizing deficits: items1,4,9,11,12,13,14 (explaining 32.5% of the variance)
- Social anxiety: items 3,5,6,8 (explaining 8.5% of the variance)
- Sensory reactivity: items 2,7,10 (explaining 6.4% of the variance)

Median score for different populations:

- **32** in 135 adults with normal intelligence diagnosed with autism spectrum disorder (ASD).
- **15** in 344 adults with normal intelligence diagnosed with attention deficit hyperactivity disorder (ADHD).
- **11** in the collapsed group of 164 adults with other psychiatric disorders, specified as psychotic disorder (n=30), mood disorder (n=59), anxiety disorder or OCD (n=67) and borderline personality disorder (n=39). The sums of reported diagnoses exceed the number of patients in the group because of co-occurring diagnoses.
- **3** in 590 non-psychiatric controls

A cut-off score of 14 or above reached a sensitivity of 97% and a specificity of:

- 46% for the ADHD group.
- 64% for the other psychiatric disorders group (described above).
- 95% for the non-psychiatric controls.

The discriminatory power of the first five items in the RAADS-14 Screen:

- A cut-off point of 4 or greater from a maximum of 15 points yielded a sensitivity of 93% and a specificity of 45% in the ADHD sample, 49% in the other psychiatric disorders group, and 90% in the non-psychiatric sample.

RAADS-14-Screen is an abridged version of Ritvo Autism and Asperger Diagnostic Scale-Revised (RAADS-R). Cite: Eriksson JM, Andersen MJ, Bejerot S. RAADS-14 Screen: validity of a screening tool for Autism Spectrum Disorder in an adult psychiatric population. *Molecular Autism* 2013; 4:49.
